# The impact of the SARS-CoV-2 pandemic on sickness absenteeism among hospital workers

**DOI:** 10.47626/1679-4435-2022-787

**Published:** 2022-03-30

**Authors:** Larissa Garcia de Paiva, Wendel Mombaque dos Santos, Graziele de Lima Dalmolin

**Affiliations:** 1 Residência em Medicina do Trabalho, Universidade Estadual de Campinas, Campinas, SP, Brazil.; 2 2 Hospital Alemão Oswaldo Cruz, Sustentabilidade e Responsabilidade Social, SP, Brazil.; 3 Departamento de Enfermagem, Universidade Federal de Santa Maria, Santa Maria, RS, Brazil.

**Keywords:** occupational health, absenteeism, hospitals, coronavirus infections, healthcare spending

## Abstract

**Introduction:**

Worker illness and, more recently, infection by the severe acute respiratory syndrome coronavirus 2 (SARS-CoV-2) can manifest as sickness absence, considerably increasing absenteeism rates, which were already rising.

**Objectives:**

To determine the impact of the SARS-CoV-2 pandemic on sickness absence rates among hospital workers and on the costs associated with them.

**Methods:**

A cross-sectional study with 1,229 workers at a University Hospital in the South of Brazil. Data were collected from absenteeism records for the period from September 2014 to December 2020 held in the Occupational Health Service database. Data were analyzed using descriptive and inferential statistics.

**Results:**

The mean sickness absenteeism rate was 3.25% and a significant increase was observed during the pandemic (5.10%) when compared to the pre-pandemic period (2.97%) (p = 0.02). During the pandemic, the mean number of sickness absence days was 2.03 times greater and the mean daily cost increased 2.49 times. Administrative assistants had the lowest relative risk (RR) of infection (RR: 0.5120; 95% confidence interval [95%CI] 0.2628-0.9974). In turn, the nursing team (RR: 1.37; 95%CI 1.052-1.787), physiotherapists (RR: 1.7148; 95%CI 1.0434-2.8183), and speech therapists (RR: 2.7090; 95%CI 1.5550-4.7195) were at greatest risk of SARS-CoV-2 infection.

**Conclusions:**

The SARS-CoV-2 pandemic led to an increase in sickness absence among workers in a hospital setting. The nursing team, physiotherapists, and speech therapists were at greatest risk of SARS-CoV-2 infection.

## Introduction

Infections caused by the severe acute respiratory syndrome coronavirus 2 (SARS-CoV-2) emerged as a new challenge for society to cope with, bearing in mind the quantity and diversity of the clinical manifestations provoked, ranging from asymptomatic patients to severe cases and deaths.^1^ Healthcare workers constitute both the main workforce fighting against the new pathology and the principal group at risk of SARS-CoV-2 infection.^2,3^ Early diagnosis of SARS-CoV-2 infection among these workers is therefore a means both for reducing transmissibility and for maintaining an active work force.^2-5^

Worker illness and, more recently, SARS-CoV-2 infection can very often manifest as sickness absence, considerably increasing absenteeism rates, which were already rising in all countries, reaching rates as high as 30% over the last 25 years.^6,7^ Sickness absence is unplanned worker absence from work because of disease or injury and has direct implications for healthcare costs because employers are obliged to pay compensation or overtime.^7^

Sickness absence increases turnover, reduces worker morale, and interrupts the continuity of patient care, causing negative impacts on both cost and quality of the services provided.^8,9^ The cost of sickness absence is not limited to paying the wages of the sick worker who does not come to work, since it also has impacts on productivity.^8,10^

It is thus necessary to expand knowledge of the impacts of the SARS-CoV-2 pandemic on sickness absence, particularly for use by hospital administrators. Although there are several different studies demonstrating that relationships between type of work and workplace are factors related to absenteeism, there are few studies demonstrating the impact of the pandemic on the health of workers in hospital settings.^7,8,10,11^ The following research question thus emerges: What impact has the pandemic had on sickness absenteeism rates and on the costs associated with it? The objective of this study was therefore to determine the impact of the SARS-CoV-2 pandemic on the rate and consequent costs of sickness absence among hospital workers.

## Methods

This is a cross-sectional study with workers at a University Hospital in the South of Brazil, which has been administrated by the Brazilian Hospital Services Company (EBSERH) since September of 2014, after a public tender process. This constitutes the starting date for data collection. The period investigated spanned from September 2014 to December 2020. The study was approved by the Research Ethics Committee at the institution, under CAAE: 80587417.0.0000.5346, decision number: 2.969.629. The requirement for free and informed consent forms was waived because the analysis was conducted using the hospital’s Occupational Health Service database.

The population consisted of health care workers whose employment contracts with EBSERH are based on the Consolidated Labor Laws (CLT), regardless of how long they have been working at the institution.

Data were collected on workers’ sickness absence (first date off from work, reason for absence, and total number of days absent) since their induction, using a Microsoft Excel spreadsheet maintained by the hospital’s Occupational Health Service. Sociodemographic data were also collected (sex, job title, and period from induction to end of employment, where applicable). Work absence was included in the analysis regardless of whether the workers in question belonged to a high-risk group for COVID-19 or whether they were in receipt of sickness benefit from the National Social Security Institution (INSS).

The values employed to calculate expenditure were based on each job title’s basic wages, without additional remuneration (unsanitary conditions premium, dangerous conditions premium, food stamps, healthcare contributions, or additional remuneration linked to career progression). This decision was made because of the diversification of wages and the prevailing legislation on planned career progression. The currency used for all different stages of calculation was the Brazilian monetary unit (the Real). The cost of absenteeism was calculated by summing the daily wage costs according to the absent worker’s job title for the relevant period, available for consultation in the EBSERH job titles, careers, and salaries plan.^12^ The cost of absenteeism did not include the cost of absenteeism when workers were receiving sickness benefit, because in these cases EBSERH stops paying wages and sickness benefit is paid by the INSS.

The rate of absenteeism per year was calculated according to recommendations from the Sub-Committee on Absenteeism of the International Association of Occupational Health,^13^ by dividing the number of days absent in 1 year by the number of days that could have been worked. This rate takes into account the weekly number of hours worked and the worker’s job title.

The total cost of absenteeism per year was calculated using the following formula:

$$\text{ Total cost of absenteeism }=(\frac{\text{ monthly wage }}{\text{ monthly workload in hours }}\times \text{ daily workload in hours })\times \text{ days off work }$$

A descriptive analysis was conducted to demonstrate the frequencies of absenteeism by study period and job title. An analysis of measures of central tendency was also conducted, calculating mean and standard deviation (SD), and the relative risk (RR) of SARS-CoV-2 infection was estimated, adopting a 5% cutoff for statistical significance. Data were analyzed using the Statistical Package for the Social Sciences (SPSS), version 21.0, and chi-square tests and analysis of variance (ANOVA) were employed to analyze the distribution of sickness absence among different groups of professionals over the period analyzed.

## Results

During the period analyzed, from September 2014 to December 2020, 1,229 workers were employed at the hospital, 928 (75.5%) of whom were absent from work at least once. In December of 2020, 1,035 workers were employed at the hospital, so 194 had left the institution (voluntary resignation by the employees).

**Figure 1:**
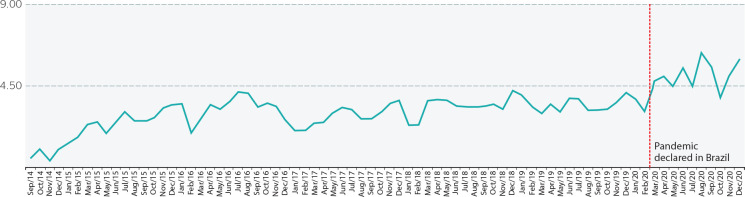
Sickness absenteeism rates by month and year, Santa Maria, RS, Brazil, 2021.

The sickness absenteeism rate was 3.25% and there was a significant difference (p = 0.01) between the rate during the pre-pandemic period (2.97%) and the rate during the pandemic (5.10%) (Figure 1).

**Figure 2:**
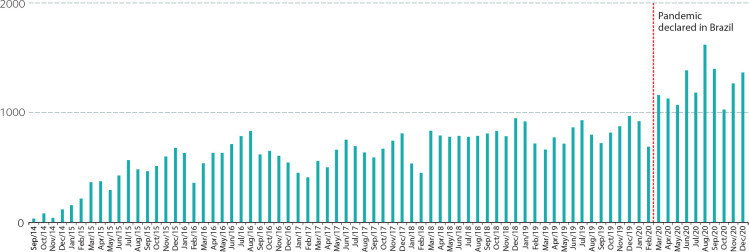
Total days of sickness absence by month and year, Santa Maria, RS, Brazil, 2021.

The mean number of sickness absence days per month during the pandemic (1,259 days) was 2.03 times greater than during the pre-pandemic period (619 days). Figure 2 shows that there were at least 1,029 days of sickness absence during every month of the pandemic.

**Figure 3:**
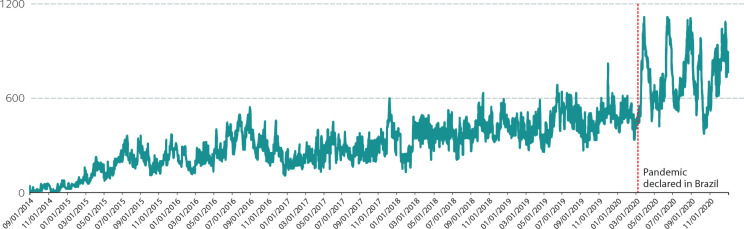
Daily cost of sickness absence, Santa Maria, RS, Brazil, 2021.

The total cost of sickness absence was R$ 8,158,117.20, with a mean daily cost of R$ 3,525.55 (SD = R$ 2,091.52). During the pandemic, the mean daily cost (R$ 7,380.38) was 2.49 times greater than during the pre-pandemic period (R$ 2,960.12) (p < 0.05). Figure 3 illustrates the daily costs of sickness absence, showing the evident increase in values exceeding R$ 6,000 per day and the wide variability during the pandemicPandemic declared in Brazil.

During the period from March to December of 2020, around 430 workers exhibited symptoms of SARS-CoV-2 infection, 43.02% (185) of whom had positive diagnoses of infection according to reverse transcription-polymerase chain reaction tests (RT-PCR). These were responsible for 3,998 days of sickness absence, equating to 31.7% of the total number of sick days during the period from March to December of 2020. The cost of sickness absence was R$ 719,964.80.

The nursing team was the group with the highest prevalence of positive cases of SARS-CoV-2 infection (61.1%), followed by the medical team (14.1%), and the physiotherapy team (6%) (Table 1). It was observed that administrative assistants had the lowest RR for infection (RR: 0.512; 95% confidence interval [95%CI] 0.263-0.997). In turn, the nursing team (RR: 1.37; 95%CI 1.052-1.787), physiotherapists (RR: 1.714; 95%CI 1.043-2.818), and speech therapists (RR: 2.709; 95%CI 1.555-4.719) had the highest risk of SARS-CoV-2 infection.

**Table 1:** Description of confirmed cases of infection by the severe acute respiratory syndrome coronavirus 2, by job title and month of infection during 2020, Santa Maria, RS, Brazil, 2021

Job title	March	April	May	June	July	August	September	October	November	December	Total
Social worker					1					1	2
Mid-level technical-administrative worker					3	2	3			1	9
Top-level administrative worker					3	1				2	6
Nurse			1	4	4	10	10	2	6	5	42
Pharmacist					1	1					2
Physiotherapist				2		5	2	2			11
Speech therapist							2		1	3	6
Physician			2	2	1	6	3		4	8	26
Nutritionist									2	2	4
Other technical-level healthcare workers			1		1	1	1		1	1	6
Nursing technician			3	7	9	16	14	4	8	10	71

## Discussion

In this study, the sickness absenteeism rate observed was approximately 3.25%, breaking down as 2.97% during the pre-pandemic period and 5.10% during the pandemic. Days absent from work because of COVID-2019 symptoms accounted for 31.7% of all absenteeism after the pandemic declaration. The increased absenteeism during the pandemic period may also be related to other sources of stress affecting the workers.^14^ The increase in patient demand, the increase in working hours, the need to wear personal protective equipment for long periods, and fear of transmitting the disease to one’s own family are all factors that contribute to the increases in burn-out and psychological stress among workers.^5-7,9,15,16^

These additional factors introduced by the pandemic must also be taken into account, going well beyond mere protection against infections.^14,17^ In this study, it was possible to observe the influence that working in a hospital setting during the pandemic had on sickness absence, since there was an overall increase in worker absenteeism (March/2020). There was clearly an increase in absenteeism of psychosocial origins, since it wasn’t until April of 2020 that the first cases of patients or workers with SARS-CoV-2 infection were confirmed at the institution studied.

The changes in hospital worker absenteeism provoked by COVID-19 were also observed in different countries.^16^ The Pan American Health Organization estimated that, up to September 2020, around 570 thousand health professionals had been infected and 2.5 thousand had died because of the pandemic in the Americas.^18^ In view of this situation, the World Health Organization (WHO) stressed that despite the challenges inherent to a new pathology still being studied, there should be no justification for worsening the standards of working conditions or for an increase in failure to comply with occupational health and safety regulations.^19,20^ In this context, preventative measures, such as testing professionals who are symptomatic or have been in contact with positive COVID-19 cases, could lead to a reduction or maintenance of the sickness absenteeism rate, keeping it at pre-pandemic percentages.

With regard to the job roles performed, the highest risks of infection were observed among the nursing team, physiotherapists, and speech therapists. This may be because of the greater viral load caused by exposure and the need to perform activities that demand direct contact with patients’ airways, which may lead to higher levels of exposure to the virus compared with other care activities. There is therefore a need for greater control of correct and regular use of personal protective equipment, because it reduces the risk of contamination by highly-infectious diseases, such as COVID-19.^17^

Also of note is the considerable economic impact of absenteeism. It was found that the mean daily sickness absence rate increased by 2.49 times in the pandemic, in comparison with the pre-pandemic period. No data were found in the literature on the cost of sickness absence during the pandemic, but prior to the pandemic developed and developing countries spent around 42% of total healthcare expenditure on remunerating the workforce, which is a little different from underdeveloped regions such as Africa and Southeast Asia.^12,21,22^ In this study, an elevated cost was observed for a small population of workers, which could indicate an elevated burden on the country’s gross domestic product (GDP), bearing in mind that in developed countries, such as those in Europe, the costs of spending on public sector workers can be as much as 2.5% of the country’s entire GDP.^8,10,23^

Historically, the nursing and medical teams account for approximately 80% of sickness absence costs, both because they are more exposed to pathologies that can be acquired in hospital settings and also because of the high numbers of these professionals compared to other workers.^24-27^ This factor impacts the work process and healthcare delivery, making it necessary to substitute the absent worker and generating additional costs for contracting replacements and/or for overtime payments.^8,11,28^

Comparing the mean annual cost, it was observed that the total had increased by approximately R$ 1,000,000 in 2020 compared with the 2 previous years (2019 and 2018). Prevention strategies could reduce the cost of sickness absence, such as vaccination campaigns, monitoring with regular tests, and implementation of standard precautions in patient care. The literature contains records of peaking costs provoked by sickness absence among healthcare workers treating influenza outbreaks, primarily in hospitals that did not run vaccination campaigns among their workers.^27-29^

The present study is subject to limitations. It was conducted at just one Federal public hospital and cannot be generalized to other Brazilian hospitals. The analysis was conservative since none of the indirect costs of these workers’ absenteeism were calculated, such as lost productivity and overtime payments to other workers, which would have led to higher cost calculations than those presented. Sums related to additional remuneration for unsanitary conditions premiums, food stamps, and career progression within the company were not considered and neither were taxes paid by the workers.

Nevertheless, this study’s originality should be emphasized, since it is the first of its kind in its evaluation of the impact of the SARS-CoV-2 pandemic on sickness absence among hospital workers and the costs related to absence. The results of this study will contribute to understanding of the pandemic in relation to maintenance of the workforce and the cost of sickness absence and its implications for healthcare.

In turn, the economic findings presented in this study demonstrate that sickness absence has a considerable financial impact and should be considered by healthcare managers. The increased demand for healthcare workers and the need for productivity demonstrate that there is a need for preemptive investment in occupational health, primarily in situations related to dealing with the pandemic that substantially increase overload. Greater action is needed for prevention, promotion, and rehabilitation in occupational health. If this is not forthcoming, the cost of sickness absence could become unmanageable for many institutions, which would compromise care for the population.

It is therefore concluded that the SARS-CoV-2 pandemic has provoked an increase in sickness absence among workers in hospital settings. Additionally, increases were observed in the direct costs linked to sickness absence and the nursing team, physiotherapists, and speech therapists were the workers at greatest risk of SARS-CoV-2 infection.
